# The Importance of Nest Box Placement for Barn Owls (*Tyto alba*)

**DOI:** 10.3390/ani12202815

**Published:** 2022-10-18

**Authors:** Motti Charter, Gabe Rozman

**Affiliations:** 1 Shamir Research Institute, University of Haifa, Katzrin 1290000, Israel; 2 Department of Geography and Environmental Sciences, University of Haifa, Haifa 3498838, Israel

**Keywords:** breeding success, nest site, sun, shade, pest control

## Abstract

**Simple Summary:**

Nest sites for cavity-breeding birds are limited in many habitats such as in agriculture. Nest boxes are artificial nest sites that are added to provide birds a place to breed in areas where natural nest sites are lacking. For example, nest boxes are used to increase barn owl numbers in agricultural areas to reduce rodent damage in farmlands. Although nest boxes are added in different locations such as on poles and trees and in sunlight/shade, it is still unknown which location is better. Here, we compared the occupation of nest boxes by barn owls on trees, nest boxes on poles in the shade, and on poles in the sun. We found that more nest boxes were occupied when they were located on trees, followed by nest boxes on poles in the shade and nest boxes on poles in the sun. The number of nestlings raised in the nest boxes varied seasonally, with more being fledged early in the season in nest boxes located on poles in the sun whereas more nestlings were fledged later in the season in nest boxes located on trees. The seasonal difference is most likely due to the lower internal temperatures in nest boxes located on trees than in nest boxes on poles in the sun. Depending on the climate, it is important to consider variation in temperatures when adding nest boxes.

**Abstract:**

Nest boxes have been used for years to increase breeding bird numbers for conservation and also in biological pest control projects. Barn owls (*Tyto alba*) have been used as biological pest control agents for rodents for years, and since nest boxes are costly for growers there is a need to determine whether nest box placement can increase the occupation of nest boxes and breeding success. We studied whether barn owl breeding in agricultural areas varied in nest boxes located on trees, poles located in the shade, and poles in the sun. The occupation of nest boxes was highest in nest boxes located on trees, followed by poles in the shade, and finally poles in the sun. In comparison, the number of fledglings was highest for nest boxes on poles in the sun followed by poles in the shade in the first half of the breeding season, whereas more nestlings were fledged in nest boxes on trees in the second part of the breeding season, which is most likely due to the higher internal temperatures in the nest boxes located in the sun. Interestingly, all the nest boxes’ internal temperatures were lower than the ambient temperatures but were much lower on trees than those on poles, most likely due to the trees providing better protection from the heat. It is therefore important to not only consider the placement of nest boxes, but how occupation and breeding success may vary seasonally.

## 1. Introduction

A lack of nest sites is one of the main limiting factors for secondary cavity-breeding bird numbers [1]. In areas where natural nest sites are lacking, nest boxes have been used to increase breeding numbers in both conservation [2] and biological pest control studies to increase natural predators that control insect [3] and vertebrate pests [4]. Barn owls have been used throughout the world as biological pest control agents of rodents in agriculture since the early 1970s in Malaysia [5], then in Israel [6,7], the USA [8,9], and other countries [10,11]. Unlike the use of smaller nest boxes for songbirds that are added to trees, buildings, or small poles, the placement of the much larger nest boxes such as for barn owls (*Tyto alba*) by farmers can be costly. The effectiveness of nest boxes may depend on where and how the farmers choose to add the nest boxes. Information on the placement of nest boxes in areas that will increase occupation and breeding success will not only save money but also increase barn owl numbers and potentially reduce rodent damage to crops. 

In most intensive agriculture there is a lack of natural nest sites due to vast open fields. The ability to place nest boxes on structures and in locations without predators and in trees [12] can greatly increase the numbers of barn owls, but these sites are often limited so many projects also add nest boxes on poles [6,13]. Adding nest boxes on poles may also be limited by locations so that the boxes will not disturb the daily work of farmers using tractors, moving irrigation, and more. 

Even though the occupation of nest boxes and breeding of barn owls in Israel have been found to be related to weather [14], variation in prey [15,16,17], movement [18], kleptoparasitic nestlings [19], and laying date [16], it is still unknown whether they are affected by the location of the nest sites (i.e., on a pole or tree). Exposure to shade versus sunlight may be important in hotter habitats, with nests receiving direct sunlight reaching higher temperatures [20,21]. Exposed nest sites may lower breeding success [22] due to increased risk of mortality [23] and fewer offspring [24,25].

To maximize the efficiency of nest boxes, it is first important to understand how nest box placement affects both the occupation and breeding success of the birds. Here we studied whether barn owl nest box occupation and breeding success differs according to the seasonal exposure to sun (shaded vs. exposed to the sun) and location (pole vs. tree) as part of a biological pest control project of rodent pests in agricultural areas in Israel. We hypothesized that nest boxes on trees would be occupied more than those on poles and that the occupation and success of nest boxes exposed to sun would decrease during the latter part of the breeding season. 

## 2. Materials and Methods

### 2.1. Study Site

We conducted this study in the Hula Valley (33°6′ N, 35°37′ E), which is mostly an intensive agricultural area (size: 31 km^2^) with crops cultivated year-round, winter crops (e.g., alfalfa, clover, garlic, oats, onion, carrot, and wheat) and summer crops (e.g., beans, cotton, corn, peas, peanuts, sunflowers, tomatoes, and watermelon).

### 2.2. Occupation and Breeding

We monitored barn owl breeding in 145 nest boxes (height = 60 cm, width = 50 cm, depth = 40 cm, entrance = 150 cm × 150 cm) on trees (N = 34), on poles in the shade (N = 35), and on poles in sun (N = 76), all located within the study site from 2012 to 2019. All nest boxes were facing east-north, were built using 15 mm birch plywood and were painted gray only on the exterior of the nest boxes. The nest boxes were added by farmers in locations specified by one of the authors (MC). All three nest types were dispersed throughout the study site equally because nest box density can affect occupation [26]. Furthermore, nest boxes do not need to be located around specific fields because barn owls have been found to hunt far from nests [27,28]. The number of monitored nest boxes varied yearly due to some boxes being damaged.

Nest boxes were visited 3–5 times between April 15 and July 30 to determine whether the nest boxes were occupied and the number of fledglings per each laying pair (pairs that laid clutches) when the oldest individual was 53 days old minus any dead nestlings found a week after fledgling. Based on years of experience researching barn owls in this study system we assume 100% detection probability, or very close to it as we have never found any owls breeding outside this period [7,26]. The Julian laying date (day first egg was laid) was determined by back-calculation, using wing length of nestlings and an incubation period of 32 d to determine age [29]. 

Precalibrated Thermochron iButtons (Dallas Semiconductor) were placed inside randomly selected nest boxes located on trees (N = 7), on poles in the shade (N = 7), on poles in the sun (N = 6), and recorded every 5 min from 16 September to 23 September 2020. We also added data loggers below nest boxes (N = 4) to determine the ambient temperature. For each day, we compared the daily maximum temperatures. We also used the mean maximum daily temperature from 2012 to 2019 (data from Israel Meteorological Center) to demonstrate how the number of nestlings in the different nest sites may be affected by external temperature. 

### 2.3. Statistics

Statistical analyses were performed with SPSS 23 and R version 4.1.1 (10 August 2021). Tests were two-tailed and *p*-values < 0.05 were considered significant. To compare occupation and the number of fledglings per successful pair, we performed generalized linear mixed-effects model (GLMM) with “year” as a random effect. The mixed effects model is an extension of generalized linear models that allows for response variables from different distributions such as, in this case, binomial with a logit link. To test the effect of nest type and laying date on probability of higher breeding success we applied an aligned rank transform test (artANOVA). The aligned rank transform allows for nonparametric testing on factorial models with fixed and random effects [30]. This test was used because Shapiro–Wilk normality test revealed the response variable significantly differed from a normal distribution (W = 0.87764, *p*-value < 0.05). Additionally, it enabled including “year” as a random effect. Finally, a one-way ANOVA followed by a Tukey post-hoc test was used to compare between temperatures of the different orientations.

## 3. Results

### 3.1. Nest Box Occupancy

**Figure 1:**
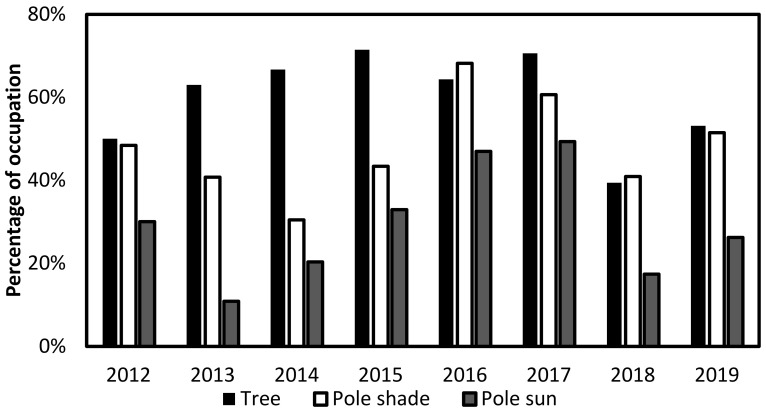
The percentage of occupation of barn owls breeding in nest boxes on trees, on poles in the shade, and on poles in the sun from 2012 to 2019.

Overall, 40.3% of the nest boxes were occupied yearly (SE = 4.1%, N = 8 years), of which 59.8% (SE = 4.0%) of the nest boxes on trees, 48.0% (SE = 4.2%) of the nest boxes on poles in the shade, and 29.2% (SE = 4.8%) of the nest boxes on poles in sun were occupied. When testing the effect that nest type has on nest occupancy, we found that the nest type significantly affects occupancy, where the probability of occupancy is significantly higher in the trees, followed by poles in the shade, and finally poles in the sun (Figure 1, Table 1).

### 3.2. Number of Fledglings 

**Figure 2:**
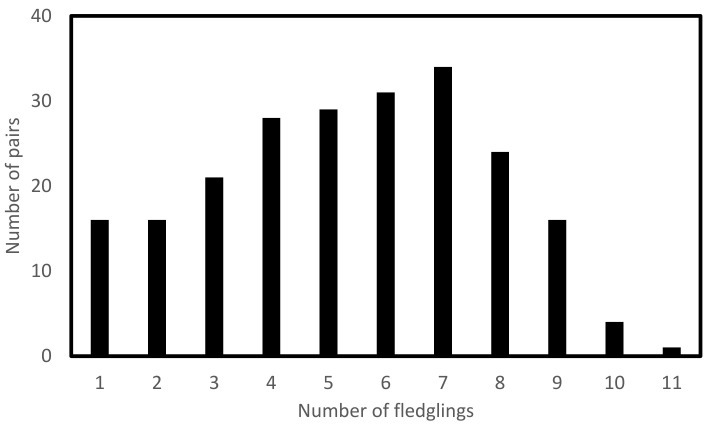
The number of nestlings that fledged per pair during the 2012–2019 breeding seasons.

**Figure 3:**
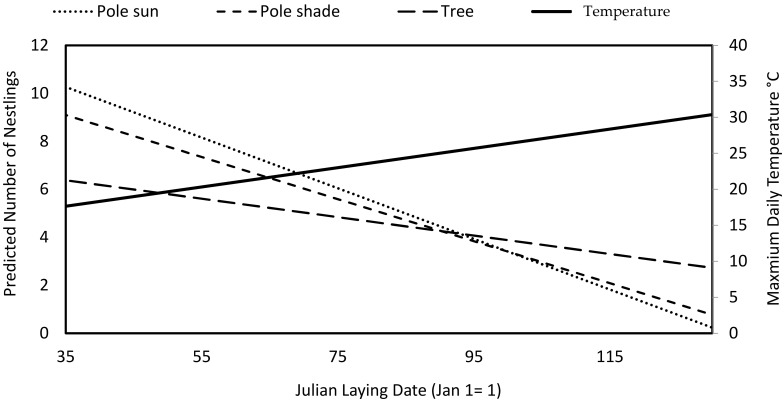
Comparison of the relationship between laying date and the number of nestlings per pair breeding in nest boxes on trees, on poles in the shade, and on poles in the sun, and the mean maximum daily temperature (solid line) from 2012 to 2019 (data from Israel Meteorological Center).

Overall, barn owls fledged 5.35 fledglings per pair (N = 220, SE = 0.16, min = 1 fledglings, max = 11 fledglings) (Figure 2) with a mean laying date of March 21 (N = 220, SE = 1.08, min = February 6, max = May 16)/Julian laying date of 81.8 (N = 220, SE = 1.08, min = 37, max = 137). Barn owl pairs therefore laid eggs over a period of 100 days. The location of the nest significantly affects the probability of having higher breeding success (Figure 3, Table 2). The number of nestlings fledged in nest boxes on poles in the sun and poles in the shade were overall significantly higher than nest boxes located on trees. The Julian laying date is significantly inversely related to the number of fledglings. The Julian laying date was significant for all interactions with type, whether the nest box was located on trees, poles in the sun, or poles in the shade. The interaction of Julian laying date with nest boxes on poles in the sun had the strongest inverse relationship with breeding success but did not significantly differ from the interaction of laying date and poles in the shade. Both interactions of laying date with nest boxes on poles in the sun and nest boxes on poles in the shade have a significantly higher inverse relationship with breeding success than the interaction of Julian laying date and the number of nestlings fledged in nest boxes on trees.

### 3.3. Temperature

**Figure 4:**
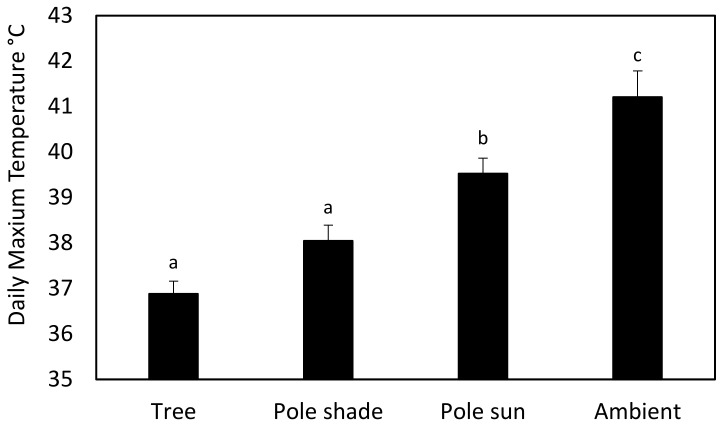
Comparison of the maximum daily temperatures (bar = standard errors) in nest boxes located on trees, on poles in the shade, on poles in the sun, and the outside temperature. Different letters indicate significant differences between nest types (Tukey multiple comparison), *p* < 0.05.

The maximum temperature varied between locations (F_3,188_ = 22.01, *p* < 0.001) (Figure 4). Nest boxes located in the shade (on trees and poles in the shade) had lower temperatures than nest boxes located in the sun. Ambient temperatures were always higher than inside nest boxes located on trees (4.3 °C higher), on poles in the shade (3.2 °C higher), and on poles in sun (1.7 °C higher).

## 4. Discussion

We found that barn owls bred in a higher percentage of nest boxes on trees than on poles located in the sun and shade. Nest boxes located on trees may have been not only protected from the elements more than those on poles but also have many branches for both the parents and later for the nestlings to perch and roost. In cases where nestlings fledge from nest boxes located on poles, the nestlings may need to fly further to find a place to roost. Even though most farmers place nest boxes within the fields in need of pest control, it is more important to add nest boxes in locations where the chances of occupation are highest (i.e., on trees, in shade, etc.) because barn owls frequently hunt over 1 km and even up to 5 km away from the nest anyways [27,28] and will therefore reach the fields with the most available rodents. Even though more nest boxes were occupied in trees than on poles, the number of nestlings fledged per pair was actually lower in the first part of the breeding season but higher in the second part. Specifically, nests on trees underperformed compared to the other nest types early in the season but performed better later in the season. This may be due to a potential cooling benefit (for fledging success) that arises most strongly for nests on trees late in the season when it is hottest. Some of the nest boxes on the poles may have been preferred due to the location of specific fields during specific years. Even though the strong effect of crop field types and other habitats on the occupancy of barn owl nest boxes has already been shown [31], future studies are needed to determine how barn owls select nest boxes and whether specific fields may affect the site selection before egg laying. Currently, this type of study may not be possible due to the limited battery life of most tracking tags and difficulties trapping adults before breeding but should be possible in the future as technologies are improving very fast. 

One of the reasons for the difference in nest box occupation may have been that the maximum temperature of nest boxes located in the shade was lower than in those in the sun and nest boxes on located on trees, potentially because of the additional protection trees provide from the elements. The internal maximum temperatures of exposed vs. shaded nest boxes in Australian woodlands were also found to be higher [32]. Maximum internal temperatures of nest boxes were found to be higher than natural cavities used by European rollers (*Coracias garrulus* L.) [33], marsh tits (*Poecile palustris*) [34], and arboreal marsupials [35]. Nest boxes located on trees had lower temperatures due to being insulated and therefore were occupied more because they are more similar to natural cavities. 

Some studies have suggested carving hollows inside trees in place of nest boxes because they were found to mimic the stable microclimates of natural cavities [36]. Although this may be true, carving hollows may not be possible because it is limited by the availability of dead trees and therefore is not possible in locations where there are no trees or where dead trees are taken out due to fears of them falling and fires. Another option that may increase occupation by decreasing temperatures is to invest in materials that will better insulate the nest boxes. Insulating nest boxes has been found to create similar temperatures to natural cavities [37]. On the one hand, insulated nest boxes may increase the stability of temperatures and the longevity of the boxes, but on the other hand, it may increase the cost per unit. Therefore, more studies are needed on different types of insulation for nest boxes to determine the economic feasibility and effectiveness for different species and regions. 

It is important to note that adding nest boxes on trees may be limited in areas that have a lot of mammalian predators. In Israel, the only climbing mammalian predators are rats, which avoid barn owl nest boxes [12] so barn owls and other bird species are able to breed without fear of nest predation. Even though the occupation of nest boxes on trees by barn owls may be higher than for nest boxes on poles, the number of trees is often limited and not found in all locations such as on agricultural land. It is therefore recommended to add nest boxes on trees where they exist and add nest boxes on poles when they do not. 

Overall, the number of nestlings fledged varied between the locations of the nest boxes, with more nestlings being fledged in nest boxes on poles than on trees. There was also an interaction between the number of nestlings and nest types and the laying date; during the first part of the breeding season, barn owl pairs that bred in nest boxes on poles fledged more offspring in the early than in the late part of the breeding season. The temperature of nest boxes located on poles exposed to the sun are significantly hotter than that of nest boxes located in the shade. Pairs that breed earlier in the season when the temperatures are lower are able to breed in nest boxes located on poles in the sun without a problem, but later on in the season, as the heat increases, the number of nestlings decrease, and it is possible that only lower quality pairs breed as shaded nest boxes are already occupied. The zone of thermal comfort (range of ambient temperature achieved without changing metabolic evaporative heat loss) of barn owl nestlings has been suggested to be up to 31 °C [38]. Nestling hatched later in the season in nest boxes on poles will therefore have more difficulties regulating body temperature which may also affect survival. Increased temperatures can cause increased nesting mortality as found in lesser kestrels (*Falco naumanni*) [23]. Furthermore, internal cavity temperatures can greatly affect the fitness of offspring [24,25]. For example, nest heat exposure can increase nestling dehydration [33]. In comparison to this study, exposed nest sites were found to have lower breeding success in African penguins (*Spheniscus demersus*) [20]. Even though earlier laying pairs can have negative impacts on fledgling success in some areas of the world, in this study site early laying barn owl pairs have higher breeding success [16], most likely because only greater quality owls are able to lay earlier even when rodent numbers are lower.

It is important to note that the maximum temperatures inside the nest boxes were lower than the ambient temperatures. Therefore, nest boxes should be able to be used in hotter climates even in direct sunlight. The suggested placement of nest boxes is not universal but should vary between climates. For example, in hotter climates such as in this study, birds selected nest boxes with lower temperatures, whereas in colder environments, birds select nest boxes that are warmer inside [39,40]. For example, in colder climates, pairs breeding in warmer cavities were found to have increased breeding success [39,41]. 

## 5. Conclusions

Barn owls occupied nest boxes located on trees more than on poles. The placement of nest boxes can increase both the occupation of the nest boxes and also breeding success of the birds, which can increase the protection of birds and also increase the efficiency of conservation and biological pest control projects. When considering the effect of temperature on the placement of nest boxes it is important to consider that many bird species lay eggs at different periods and therefore some locations may be selected only during specific parts of the year. Adding nest boxes of different types and locations will therefore increase the number of breeding pairs during the year. Future projects are needed to study whether nest box placement varies between habitat sites and regions. 

## Figures and Tables

**Table 1:** Generalized linear mixed model testing the effect of nest type on probability of occupancy of barn owls breeding in nest boxes on trees, on poles in the shade, and on poles in sun.

Variable	Estimate	SE	z Value	*p* Value
Tree (intercept)	0.407	0.206	1.977	0.048
Pole sun	−1.340	0.171	−7.827	<0.01
Pole shade	−0.491	0.196	−2.505	0.012

**Table 2:** Aligned rank transform test assessing the effect of nest type and laying date on probability of higher breeding success for laying pairs.

Variable	Estimate	SE	z Value	*p* Value
Tree (intercept)	0.739	0.095	7.750	<0.01
Pole in the shade	0.478	0.185	2.579	0.010
Pole in the sun	0.571	0.133	4.293	< 0.01
Tree: laying date	−0.004	0.001	−3.604	<0.01
Pole in the shade: laying date	−0.006	0.002	−2.568	0.010
Pole in the sun: laying date	−0.006	0.002	−3.904	<0.01

## Data Availability

Not applicable.
